# Evaluation of Anti-*Candida* Potential of *Piper nigrum* Extract in Inhibiting Growth, Yeast-Hyphal Transition, Virulent Enzymes, and Biofilm Formation

**DOI:** 10.3390/jof8080784

**Published:** 2022-07-27

**Authors:** Claudia Patrícia Bravo-Chaucanés, Yerly Vargas-Casanova, Luis Carlos Chitiva-Chitiva, Andrés Ceballos-Garzon, Geison Modesti-Costa, Claudia Marcela Parra-Giraldo

**Affiliations:** 1 Unidad de Proteómica y Micosis Humanas, Grupo de Enfermedades Infecciosas, Departamento de Microbiología, Facultad de Ciencias, Pontificia Universidad Javeriana, Bogotá 110231, DC, Colombia; claub06@gmail.com (C.P.B.-C.); y.vargasc@javeriana.edu.co (Y.V.-C.); andress1224@hotmail.com (A.C.-G.); 2 Grupo de Investigación Fitoquímica Universidad Javeriana (GIFUJ), Departamento de Química, Facultad de Ciencias, Pontificia Universidad Javeriana, Bogotá 110231, DC, Colombia; chitival@javeriana.edu.co (L.C.C.-C.); modesticosta.g@javeriana.edu.co (G.M.-C.); 3 UMR 0454 MEDIS, INRAE, Université Clermont Auvergne, 63000 Clermont-Ferrand, France

**Keywords:** *Piper nigrum*, *Candida albicans*, *C. auris*, morphogenesis, antibiofilm, enzyme activity, toxicity

## Abstract

Due to the increased incidence of fungal infections and the emergence of antifungal resistance mainly by *Candida* species, the need for safe and effective novel therapies is imperative. Consequently, plants and herbs are a powerful source to combat infections. Here, we evaluated the anti-*Candida* potential of an ethanolic extract from *Piper nigrum.* The phytochemical analysis of *P. nigrum* revealed bioactive compounds such as alkaloids, terpenoids, and tannis. Our results showed that *P. nigrum* extract suppressed the virulence factors of *C. albicans* strains, including hyphae formation in both liquid and solid media, reduced secretion of phospholipases/proteinases, and affected biofilm formation. Furthermore, the *P. nigrum* extract showed no hemolytic effect in vitro and exhibited reduced cytotoxicity on Vero cells and *G. mellonella* larvae at concentrations that inhibited hyphae and biofilm in *C. albicans.* Moreover, the extract demonstrated antifungal activity against *C. auris* strains. In conclusion, the *P. nigrum* extract affected the growth and morphogenesis of *Candida* (even in resistant strains), demonstrating that this plant has an anti-candida activity and represents a promising resource for discovering novel antifungal compounds.

## 1. Introduction

*Candida* species represent the most common cause of invasive fungal infections (IFIs), particularly in immunocompromised patients [1], *Candida albicans* being the most frequently isolated of them. This fungus, which co-exists as a commensal organism of the skin, the vaginal and oral cavities, and gastrointestinal microbiota, usually causes no harm in healthy individuals, as its growth is restrained by the immune barriers and other residents of the microbiota [2,3]. However, it will likely change when tissue homeostasis is disrupted [4], causing superficial but more severe, life-threatening systemic infections [5]. The associated mortality is about 50% [6,7]. In general, the poor prognosis is partly attributable to the limited antifungal armamentarium, i.e., azoles, polyenes, and echinocandins, that exhibit disadvantages such as side effects, toxicity, and complex drug interactions, and the ongoing problem of antifungal resistance [8,9,10]. Due to these limitations, current research has focused on developing alternative therapies for treating *Candida* infections [7,11,12].

The pathogenicity of *Candida* spp. derives from multiple factors, including its high ability to adapt to stressful conditions [13,14]. Moreover, virulence attributes such as the expression of surface molecules as adhesins, the ability to change its morphology, the capacity to form a biofilm, and the secretion of hydrolytic enzymes are essential for establishing infection [15,16]. Therefore, counteracting these traits could lead to the development of new therapies. Depending upon the environmental conditions, *C. albicans* may grow either as unicellular yeast cells (blastospore), as elongated ellipsoid cells with constrictions at the septa (pseudohyphae), or true hyphae [17]. This reversible morphological transition triggers a transcriptional increase leading to the activation of multiple essential pathways for *C. albicans* virulence [18]. Many authors have suggested that the virulence of *C. albicans* could be attributed -partially, at least- to the filamentous form following a yeast-hyphal transition [19,20]. Blastospore cells are well suited to dissemination upon infection, as well as hyphal cells to host invasion and tissue damage [21,22]. This transition includes extensive changes in gene transcription, cell wall structure, and virulence trait expression [16,19,23]. Among the extracellular enzymes, phospholipases and proteinases play a vital role because they are involved in adherence, tissue penetration, and invasion of the host [24].

Infections resulting from the formation of biofilms, if unsuccessfully managed, can have devastating consequences since microbial communities can colonize devices such as catheters, dentures, and prosthetic joints. In addition, biofilms can tolerate extremely high concentrations of antifungals. The impact on public health is dramatic as cells released from biofilms can migrate into the bloodstream and cause systemic infections, which carry high morbidity and mortality [25,26]. According to Jacobsen and coworkers, inhibition of yeast-to-hyphal transition could be an important option for controlling *C. albicans* infections, prophylactically and as a treatment [19].

Another concerning species is *C. auris*, a multi-drug resistant yeast first reported as a human pathogen in 2009 in a female Japanese patient. Henceforth, it has been reported in over 40 countries on five continents, including Colombia, with reports made by our group [27,28]. Although an accurate mortality rate is unknown, it might be said that about 30–60% of patients who contracted *C. auris* systemic infection have died [29,30]. In addition, as with other *Candida* species, *C. auris* produces biofilms that commonly decrease susceptibility to antifungals [31]. Additionally, delays in diagnosis constitute another difficulty in treating *C. auris*, as this pathogen is not included in library databases in many clinical diagnostic systems [32,33].

New sources for the development of antifungal drugs are under investigation, being plant-derived compounds promising ones [34,35,36]. For instance, several plant extracts have been reported to have anti-*Candida* activities, including *Allium sativum* (Garlic), *Cinnamomum verum* (Cinnamon), *Origanum vulgare* (Oregano), *Salvadora persica* (Souwak), and *Ziziphus spina*, among others [37,38,39,40,41]. Furthermore, some studies have explored plant-derived molecules against *Candida* biofilms, showing encouraging results [34,42]. The diversity of natural products and their multiple chemical structures have attracted considerable attention, given that some compounds offer certain advantages in terms of efficiency and selectivity of targets [43]. Interestingly, *Piper nigrum* (black pepper) has been used for many purposes as a natural medicinal agent for the treatment and alleviation of influenza, migraine, and strep throat, among others [44]. *P. nigrum*, has a variety of active metabolites that act as a defense system against various pathogenic agents [45]. Additionally, a recent study highlights the antioxidant, antibacterial and antimutagenic potential of *P. nigrum* seeds extracts [46]. Antifungal activity against *Candida* spp. has also been demonstrated, but its effect on virulence factors and against antifungal-resistant clinical isolates has been barely evaluated [47,48].

This study aimed to evaluate the effect of a crude extract from *P. nigrum* against *C. albicans* susceptibility, yeast-hyphal transition, virulent enzyme production, and biofilm formation. Additionally, we assessed the toxicity of *P. nigrum* extract using Vero cells in vitro and *Galleria mellonella* larvae model in vivo. Finally, the effect on susceptibility and biofilm development was evaluated in clinical isolates of *C. auris*.

## 2. Materials and Methods

### 2.1. General Experimental Procedures

The extraction process was carried out using 96% ethanol solvent. Thin layer chromatography (TLC) studies were performed using silica gel 60 F_254_ chromatographic plates purchased from Merck^®^ (Darmstadt, Germany). The reference standards used: berberine, kaempferol, gallic acid, and lupeol, were acquired from Sigma Aldrich (St. Louis, MO, USA). For UPLC-PDA chromatographic analysis, HPL grade MeOH, ACN, H_2_O, and formic acid (FA), the reference standards were purchased from Merck^®^ (Darmstadt, Germany).

### 2.2. Plant Material and Extract Preparation

The fruits of the species *P. nigrum* were collected in April 2019 during the vegetative stage in the municipality of Orito, Putumayo, in Colombia; geographic coordinates: 0°37′45″ N, 76°51′55″ W. The taxonomic determination of the species was carried out by the biologist Néstor García in the herbarium of the Pontificia Universidad Javeriana with the collection number HPUJ-30548. The plant material (80 g) was dried in an oven with circulating air at 40 °C for 96 h for subsequent grinding in a blade mill. The dried and ground plant material was extracted by percolation with EtOH 96% in a ratio of 1:10 (*w*/*v*), at room temperature, protected from light in 4 cycles of 24 h each (solvent changes). The extracts from the different cycles were pooled and concentrated under reduced pressure by rotary evaporation at a temperature not exceeding 40 °C. They were stored at room temperature in amber vials duly labeled for later analysis. A 10 g dry extract was obtained and fractionated by Vacuum Liquid Chromatography (VLC) using solvents of increasing polarity: Hexane (1 g), Dichloromethane (DCM) (2.8 g), Ethyl Acetate (EtOAc) (2.5 g) and Ethanol–Water (EtOH–H_2_O) 80:20 (*v*/*v*) (3.7 g).

A stock solution of *P. nigrum* ethanolic extract was dissolved in dimethyl sulfoxide (DMSO) at a final concentration of 60 mg/mL and stored at −20 °C. In all experiments, a DMSO control was included.

### 2.3. Phytochemical Analysis

The HPTLC analysis was performed using CAMAG^®^ brand HPTLC, consisting of an autosampler (ATS 4), developer (ADC), derivatizer (DV), development chamber, visualization chamber, and VisionCATS software. From each sample, 10 µL of a solution at a concentration of 10 mg/mL in EtOH were applied as a band on 20 × 10 cm Merck^®^ HPTLC Silica gel 60 F_254_ plates, which were eluted with different mobile phase systems according to the polarity characteristics of the corresponding metabolites found (Figure S1). The developers used were: Dragendorff’s Reagent (alkaloids), NP-PEG Natural Reagent (flavonoids), 1% FeCl_3_ Reagent (tannins), and *p*-anisaldehyde sulfuric reagent (triterpenes).

The chromatographic analyses were performed using ACQUITY H-Class equipment. The UPLC system was managed by Empower^®^ 3 Chromatography Data (Waters Corporation, Milford, MA, USA). The detector was an ACQUITY UPLC photodiode array (PDA). A Phenomenex^®^ Kinetex EVO C18 column (100 × 2.1 mm, 2.6 µm, 100 Å) was used at 30 °C with an elution gradient of water in 0.1% formic acid (solvent A) and acetonitrile (solvent B) as follows: 3% B for 0 to 3 min, 3 to 95% B for 3 to 30 min, 95% B for 30 to 32 min, 95 to 3% B for 32 to 35 min, and 3% B for 35 to 40 min, with a run flow rate of 0.40 mL/min and an injection volume of 2 µL. For detection, the wavelength used was 200 to 400 nm. Mass spectrometric (MS) fraction analysis was performed on LC-MS QqQ 9030, Nexera X2 (Shimadzu, Duisburg, Germany) system. The ionization method was ESI operated in positive ion mode. The fraction was prepared at a concentration of 1000 µg/mL, and an injection of 2 µL was performed for analysis.

### 2.4. Fungal Strains

The reference strain *C. albicans* SC5314 was used in most phenotypic assays. In addition, one *C. albicans* fluconazole (FLC)-resistant isolate (CAAL256) was included to evaluate the effect of the ethanolic *P. nigrum* extract. Likewise, two clinical isolates of *C. auris* FLC-sensitive (CAAU435) and FLC-, amphotericin b (AMB)-resistant (CAAU537), previously characterized and identified (MALDI TOF-MS) in our group, were used in some experiments [28,49].

### 2.5. In Vitro Antifungal Susceptibility Test

Antifungal susceptibility testing was carried out using the broth microdilution method (BMD), following the CLSI M27-A3 guidelines with slight modifications [50]. Briefly, yeast suspension was made in 0.85% saline solution (SS) and adjusted at 1–5 × 10^6^ cells/mL (0.5 McFarland standard). Then, it was diluted in liquid RPMI 1640 medium (Sigma-Aldrich, Saint Louis, MA, USA) (with MOPS, pH 7.2) and adjusted at 0.5 × 10^3^–2.5 × 10^3^ cells/mL. One hundred μL of yeast inoculum was added to a 96-well plate containing serial 2-fold dilutions of the extract. The final concentrations of the ethanolic *P. nigrum* extract used ranged from 4 to 2048 μg/mL. FLC drug was used as a control (0.125 to 128 μg/mL).

Minimal inhibitory concentrations (MICs) were visualized, and densitometry (595 nm, microplate reader, iMarK^TM^, Bio-rad) was used to determine the lowest concentration of the ethanolic extract that caused a significant decrease compared with that of the extract-free growth control after 48 h of incubation. The MIC endpoint was defined as the lowest concentration of *P. nigrum* ethanolic extract able to inhibit 80% of the cell growth compared to its respective positive control (extract-free). Three independent assays were performed.

To verify that the extract could kill the yeast cells, the plates were also evaluated for minimum fungicidal concentration (MFC). Briefly, aliquots from each well from susceptibility testing assays were transferred to plates containing Sabouraud Dextrose Agar (SDA), which were then incubated at 37 °C for 24 h. The highest dilution with no growth on the agar plate was MFC [51].

### 2.6. Time-Kill Kinetic Assay

Time-kill kinetic assay was carried out according to the method previously described by Pfaller and coworkers [52] with minor modifications. Briefly, yeast cells from an overnight culture were grown in SDA agar at 37 °C and adjusted spectrophotometrically (0.5 McFarland turbidity standard at 595 nm) to a final concentration of 10^3^ cells/mL in RPMI-1640 medium (with MOPS, pH 7.2). Then, 150 μL of cell suspension was added to a 96-well plate containing serial 2-fold dilutions of the extract, ranging from 16 μg/mL to 4096 μg/mL. Plates were loaded into a Bioscreen C equipment at 37 °C under constant agitation. One-hour interval measurements were taken at 600 nm. Readings were analyzed with Bioscreen software (Growth Curves USA, Piscataway, NJ, USA). For determining the fungistatic and fungicidal effects, a growth inhibition >50% for 48 h was considered fungistatic, and a yeast kill of ≥99% for 72 h was considered fungicidal.

### 2.7. Yeast-to-Hyphal Transition Test

To assess the effect of *P. nigrum* ethanolic extract on the yeast-to-hyphae transition of *C. albicans*, two transition-inducing media were used: RPMI 1640 and yeast extract peptone dextrose (YPD) supplemented with 10% fetal bovine serum (FBS) as previously described [53,54], with some modifications. Precisely, *C. albicans* strains (SC5314 and CAAL256) were grown overnight in broth fresh YPD media at 30 °C with shaking. Two-milliliter aliquots from the overnight cultures were centrifuged at 5000× *g* rpm for 10 min and washed twice with an equal volume of phosphate-buffered saline (PBS) at pH 7.2. The washed cells were resuspended in an equal volume of PBS and adjusted to an inoculum of 1–5 × 10^6^ cells/mL. Afterward, 200 μL of cells were added to 1 mL of RPMI 1640 medium (RPMI; Gibco, Grand Island, NY, USA) with 2.1 mM L-glutamine and buffered with 165 mM MOPs (pH 7.0), or 1 mL of YPD medium supplemented with FBS. Both transition-inducing media contained 8–512 µg/mL of *P. nigrum* ethanolic extract, and their fractions (isolated compounds) (Hexane, DCM, EtOAc, and EtOH:H_2_O) were investigated in germ tube formation to identify which active compound affects this virulence attribute. Tubes were incubated with shaking (200 rpm) for 4 h at 37 °C under aerobic conditions. Blastopore and hyphal forms (250 per sample) were examined under an inverted microscope (ICX41 coupled with an OD400UHW-P digital microscope camera at ×40 magnification). The RPMI and YPD + FBS transition-inducing media without *P. nigrum* extracts and non-inducing culture (only YPD media) were used as controls [55].

Yeast-to-Hyphal transition was also induced in solid media [56]. Briefly, 50 μL of *C. albicans* cell suspension (1 × 10^6^ cells/mL) were spread on non-inducing (YPD) and inducing YPD agar plates (10% FBS, 10 g yeast extract, 20 g peptone, 2% dextrose) without (control) or with the addition of different concentrations of the *P. nigrum* ethanolic extract. The plates were incubated for 7 days at 37 °C; then, the morphology of the fungal colonies was recorded by microscopy at 40× magnification.

### 2.8. Biofilm Formation Assay

The effect of *P. nigrum* ethanolic extract on the biofilm formation of *C. albicans* strains was tested in 96-well microplates [57,58]. One hundred µL of *Candida* cell suspensions (1 × 10^6^ cells/mL) in RPMI-1640 with MOPS were dispensed in 96 microdilution wells with or without *P. nigrum* extract (8 to 2048 μg/mL). The plates were then incubated at 37 °C and allowed to adhere for 1 h. The non-adherent cells were removed, and 200 μL of fresh RPMI was added. The plates were incubated further at 37 °C for 24 h. After incubation, biofilms were washed twice with PBS, and finally, 200 µL of RPMI-1640 plus 10 µL of 700 µM resazurin (Sigma–Aldrich) was added to each well and incubated at 37 °C for 2 h. The biofilm was quantified indirectly by measuring the fluorescent water-soluble resorufin product that results when resazurin is reduced by reactions associated with respiration. Fluorescence was measured at 560 nm with emission at 590 nm in an automated plate reader (Model 550 Microplate Reader Bio-Rad, Milan, Italy). Caspofungin (0.03 to 1 μg/mL) was used as a standard antifungal drug.

### 2.9. Phospholipase and Proteinase Production

Both the production of phospholipase and proteinase were evaluated in *C. albicans* strains after *P. nigrum* ethanolic extract exposure. For phospholipase production, *Candida* cells were grown overnight at 37 °C. The cells were then suspended in PBS and adjusted at 1–5 × 10^6^ cells/mL and exposed to the *P. nigrum* ethanolic extract at 32–512 μg/mL. Cell suspensions were incubated at 37 °C with shaking (80 rpm) for 2 h. After incubation, the cells were washed twice and suspended in PBS. Five μL (treated or untreated with the extract) were spotted in an egg-yolk agar medium (13 g SDA, 11.7 g NaCl, 10% sterile supernatant of egg yolk emulsion, and 184 mL distilled water). The plates were incubated at 37 °C, and the results were recorded after 5 days [59].

The precipitation zones (*Pz*) around the yeast colonies were measured to determine the enzyme activity in millimeters. The *Pz* coefficient were transformed into scores: *Pz* 1.0 (−) indicates that the tested strain was negative for phospholipase production, 0.99–0.90 (+) very low; 0.89–0.80 (+ +) low; 0.79–0.70 (+ + +) moderate producers; and *Pz* ≤ 0.69 (+ + + +) high producers, which means this species releases large amounts of enzymes (strongly positive) [60,61].

Proteinase activity was assessed according to the method described by Kantarcioglu and Yücel [62], with slight modifications. The test medium consisted of agar plates containing bovine serum albumin (BSA) (fraction V) 0.5 g, and 60 mL of a solution containing 0.04 g MgSO_4_·7H_2_O, 0.5 g K_2_HPO_4_, 1 g NaCl, 0.2 g dried yeast extract, and 4 g glucose, pH 5. This solution was sterilized by filtration and mixed with 140 mL of agar. After 7 days of incubation at 37 °C, the reaction was stopped by adding 10% trichloroacetic acid (sigma-aldrich), and the dishes were stained with 0.5% Coomassie Brillant Blue G-250. The diameter of the unstained zones around the colonies was considered a measure of proteinase production. The calculated *Pz* was used to categorize the strains as mentioned above.

### 2.10. Hemolytic Activity Assay

The hemolytic activity of the ethanolic extract of *P. nigrum* was measured as the amount of hemoglobin released by the lysis of human erythrocytes [63]. Briefly, 5 mL of peripheral blood from a healthy individual was collected in an EDTA tube and centrifuged at 1000× *g* for 7 min. The erythrocyte-rich fraction was suspended in PBS and washed twice by centrifugation at 1000× *g* for 7 min. Then, 100 μL of the *P. nigrum* extracts (16–2048 μg/mL) were mixed with 100 μL of erythrocytes (4% hematocrit) in round-bottom 96-well plates and incubated at 37 °C for 2 h. Subsequently, the reaction was stopped by centrifuging for 10 min at 500× *g*. The supernatant (100 μL) containing the hemoglobin released from lysed erythrocytes was transferred to a flat-bottom 96-well plate to measure hemoglobin (540 nm). PBS was used as a negative control, while Tween-20 (20% *v*/*v*) in PBS was used as a positive control. The DCM (dichloromethane) fraction from ethanolic *P. nigrum* extract with the higher proportion of the principal alkaloid, piperine, was also used to evaluate anti-hemolytic activity against human erythrocytes.

### 2.11. Cytotoxicity Assay

The *P. nigrum* extract was tested for in vitro cytotoxicity using Vero cells by 3-(4,5-dimethylthiazol-2-yl)-2,5-diphenyltetrazolium bromide (MTT) assay [64]. Vero cells were seeded in a 96-well plate at a density of 2.5 × 10^4^ cells/well and allowed to adhere and proliferate for 24 h in DMEM (Dulbecco’s Modified Eagle’s) medium. Subsequently, the ethanolic *P. nigrum* extract was prepared (8–168 μg/mL) using DMEM medium as a solvent. Extract solutions were added to wells containing the adhered cells. Then, plates were incubated for 24 h at 37 °C under a 5% CO_2_ atmosphere. After incubation, the medium was removed, the cells were washed with PBS, and 30 μL of MTT was added (1 mg/L in PBS). The cells were incubated at 37 °C for 4 h; after that, MTT was removed, and 100 μL of DMSO was added to solubilize formazan crystals, resulting from the metabolism of MTT. Cell viability was estimated by measuring the absorbance of formazan at 490 nm using the BioTek ELx800 absorbance reader. In addition, the half-maximal inhibitory concentration (IC_50_) was calculated by plotting viability versus log (concentration).

### 2.12. In Vivo P. nigrum Toxicity

A toxicity assay was assessed in a *Galleria mellonella* model described by Moya-Andérico and coworkers [65]. Briefly, the larvae were obtained from Productos Biológicos Perkins Ltda (Palmira, Valle del Cauca, Colombia). Larvae in the last larval stadium weighing 250–330 mg and with a length of approximately 2 cm were selected. The larvae were submerged in 0.1% sodium hypochlorite solution for 30 s and rinsed with sterile deionized water. Groups of 10 were injected with a 0.5 mL (BD^®^) gauge insulin syringe through the last left pro-leg using a 10 μL inoculum of various concentrations of *P. nigrum* extract. The control group was inoculated with 10 μL of 1× PBS or DMSO. After inoculation, the larvae were placed in Petri dishes and incubated in darkness at 37 °C. The dead larvae were detected by a color change (dead individuals become dark brown) and lack of movement. The number of dead larvae was recorded daily [65].

### 2.13. Statistical Analysis of Data

All experiments were performed on three independent biological replicates. The effect of *P. nigrum* extract on *Candida* transition, phospholipase, and proteinase production during biofilm formation was analyzed using the ANOVA and Dunn’s multiple comparison test. The hemolytic activity and MTT assay were analyzed using a one-way ANOVA test. Survival curves were constructed using the method of Kaplan and Meier; then, the curves were compared using the Log-Rank (Mantel-Cox) test. The data were expressed as means ± standard error of the mean (S.E.M.). The significance was considered at *p* values of <0.05. The statistical models were constructed and analyzed using GraphPad software (version 7) (GraphPad Software Inc., La Jolla, CA, USA).

## 3. Results

### 3.1. Phytochemical Analysis

The highest yield of the fractionation performed by VLC was obtained in the EtOH-H_2_O fraction with 37%, followed by the DCM, EtOAc, and Hexane fractions with 28, 25, and 10%, respectively.

The HPTLC fingerprint analysis of the ethanolic extract of *P. nigrum* allowed us to determine the presence of different groups of secondary metabolites, the major compounds being of the alkaloids type. The presence of other groups of compounds corresponding to tannins and terpenoids reported in the literature [66] was observed (Figure S1). The analysis of the chemical profile by this technique and using Dragendorff’s reagent as a specific derivatizer allowed us to detect the presence of a major compound presenting an orange band corresponding to compounds of alkaloid type. The fractions obtained were not analyzed by HPTLC; only the extract of the fruits of *P. nigrum* was analyzed regarding its total chemical composition.

**Figure 1:**
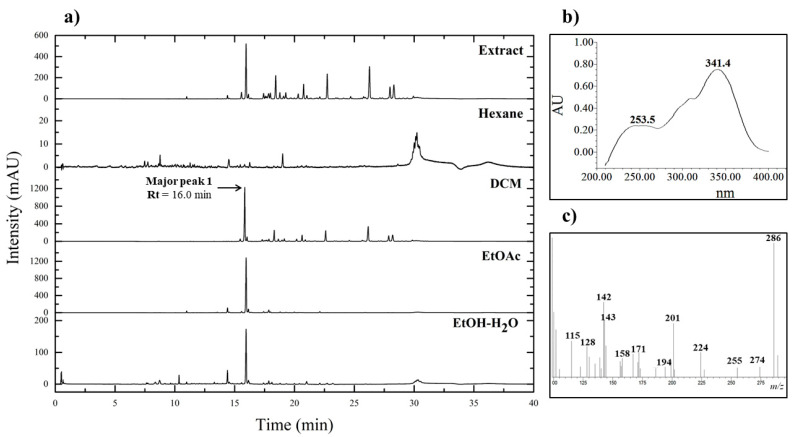
(**a**) Chromatograms corresponding to crude extract and fractions from fruits of *P. nigrum* obtained by UPLC-PDA at wavelength 260 nm. (**b**) UV adsorption spectrum of major peak 1 with Rt of 16.0 min. (**c**) MS spectrum of major peak 1 with Rt of 16.0 min.

The results of chromatographic analysis by UPLC-PDA of the crude extract and fractions showed that the chromatographic profile of the ethanolic extract of *P. nigrum* contains a great variety of peaks corresponding to compounds of medium to low polarity as observed in the wavelength evaluated at 260 nm (Figure 1a). It was found that the peak denominated as 1 with a retention time (Rt) of 16.0 min is found in greater proportion concerning the other compounds presented by the extract and the fractions of the fruits of *P. nigrum,* respectively. The four fractions obtained (Hexane, DCM, EtOAc, and EtOH–H_2_O) were analyzed to compare them with the crude extract of *P. nigrum*.

The major peak 1 is present in the DCM, EtOAc, and EtOH:H_2_O fractions, where the largest area is in the DCM fraction. From the analysis, it was found that the DCM fraction has the highest number of peaks corresponding to the compounds present. In this regard, an adequate fractionation was observed, taking into account the distribution of the compounds in each fraction according to their polarity.

The chromatographic system used in this study provided sufficient separation of the compounds present in the extract. The resulting major peak **1** in the chromatogram was checked to identify the putative compound based on the full UV spectrum recorded by the PDA detection system (Figure 1b). The identification of the major peak was done by comparing the UV spectrum with the spectra obtained in previous research conducted on the fruit extract [67]. According to the results, the major compound of the DCM fraction presented two adsorption bands at 253.5 and 341.4 nm.

Mass spectrometry analysis of the DCM fraction was performed to determine the fragmentation patterns of Major peak 1. It was also used to perform a structural characterization of the putative compound present. The results of this analysis permitted the determination of the mass spectrum of Major Peak 1; different characteristic fragmentation patterns were found in *m*/*z* 286, 201, 171, 143, and 115 (Figure 1c).

### 3.2. Effect of P. nigrum on C. albicans Growth

The impact of the ethanolic extract of *P. nigrum* on the growth of *C. albicans* was initially investigated by the determination of MIC and MFC. Results revealed an equal MIC and MFC value of 2048 μg/mL for both *C. albicans* strains (SC5314 and CAAL256), indicating that the growth was affected by the extract. In addition, the activity of Hex (n-hexane), DCM (dichloromethane), EtOAc (ethyl acetate), and EtOH:H_2_O (Ethanol–Water) fractions were evaluated. The MIC and MFC values were superior to the total extract, which had been found on screening to be inactive against *C. albicans* strains (Figure S2).

**Figure 2:**
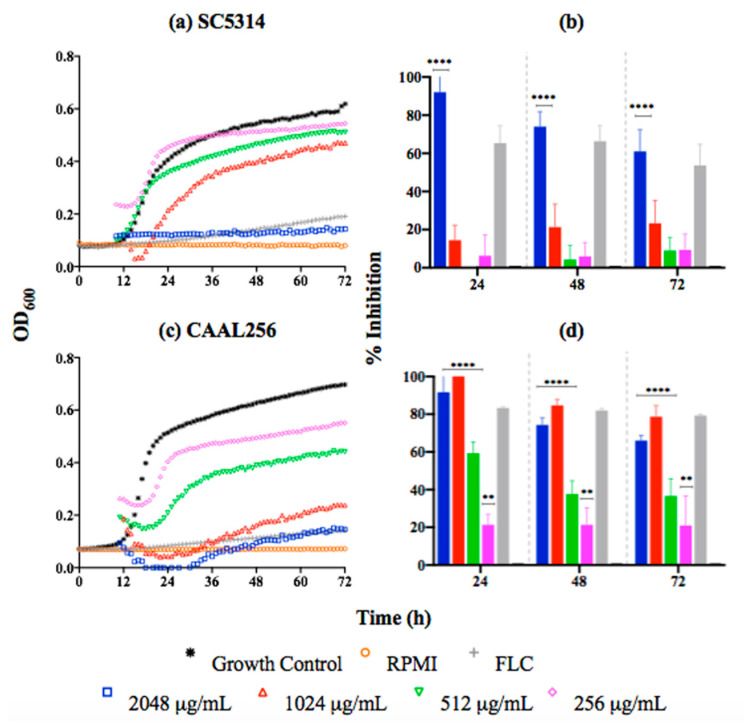
Time-kill kinetic curves of *C. albicans* strains exposed to *P. nigrum* ethanolic extract; (**a**) SC5314 kinetic growth (FLC concentration, 1.0 μg/mL), (**b**) percentage of inhibition. (**c**) CAAL256 kinetic growth (FLC concentration, 64 μg/mL), (**d**) percentage of inhibition. *p*-values of <0.05 were used to indicate statistical significance as follows: **** *p* < 0.0001, ** *p* < 0.0068.

Regarding the kinetic results, the curves indicate that the *C*. *albicans* SC5314 strain in the presence of *P. nigrum* at 2048 μg/mL (MIC) was significantly inhibited (60–90% after 72 h of growth) (Figure 2a,b). For the FLC-resistant CAAL256 strain, concentrations from 256 μg/mL induced significant growth inhibition. However, percentages of inhibition higher than 60% were only observed from the concentration of 1024 μg/mL. Nonetheless, that result represents a greater antifungal activity than the reference strain (Figure 2c,d).

### 3.3. Effect of P. nigrum against C. albicans on Yeast-to-Hyphal Transition

**Figure 3:**
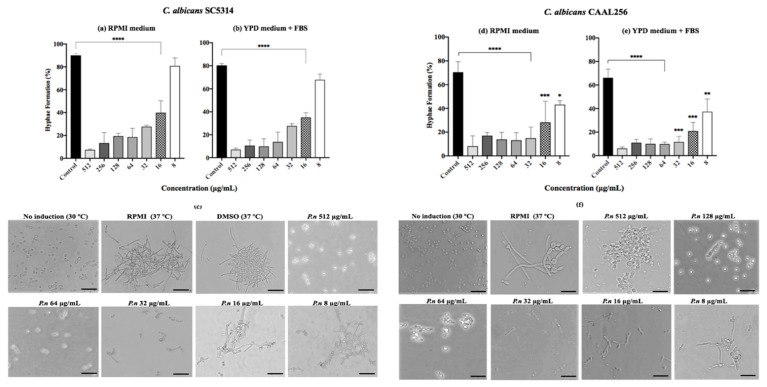
Effect of *P. nigrum* ethanolic extract treatment on yeast to hyphal transition. Percentage of filamentation in *C. albicans* (**a**,**b**) SC5314 and (**d**,**e**) CAAL256 strains, photographic record of (**c**) SC5314 (**f**) CAAL256. The scale at the bottom right of the images represents 50 µm. *p*-values of <0.05 were used to indicate statistical significance as follows: * *p* ≤ 0.05. ** *p* ≤ 0.01. *** *p* ≤ 0.001. **** *p* ≤ 0.0001.

Since yeast-to-hyphal transition represents one of the key traits of *C. albicans,* we evaluated the effect of *P. nigrum* on this switching process. Results showed that the transition to germ tubes was significantly (*p* < 0.001) reduced in SC5314 and CAAL256 strains treated with the ethanolic extract of *P. nigrum* (16–512 μg/mL) in a dose-dependent manner after 4 h of incubation in both RPMI-1640 medium and YPD medium supplemented (Figure 3). Considering the results of strain SC5314, moderate hyphal growth and germ tube formation were observed at 16 μg/mL. However, the extract loses its inhibitory effect at 8 μg/mL, and an entangled hyphal network was observed.

We also observed that the effect of the ethanolic extract is maintained in the FLC-resistant strain CAAL256, given that Yeast-to-hyphal transition was considerably affected in all concentrations tested. For instance, at 32 μg/mL, the extract reduced CAAL256 hyphae formation by more than 50% using both inducing media (Figure 3d,e). Notably, a low number of cells developed true hyphae, while in the control group of CAAL256, germ tubes/pseudohyphae and clusters of cells were found; in contrast to the SC5314 strain, where mainly true hyphae and large aggregations of cells entangled by hyphae were regularly observed (Figure 3c–f).

Interestingly, when the yeast-to hyphal transition in *C. albicans* SC5314 was evaluated using other extract fractions, the DCM maintained hyphal transition suppressed even at 8 μg/mL (*p* < 0.05), while Hex, EtOAc, or EtOH:H_2_O fractions lost their inhibitory capacity between 32 to 64 μg/mL (Figure S3).

**Figure 4:**
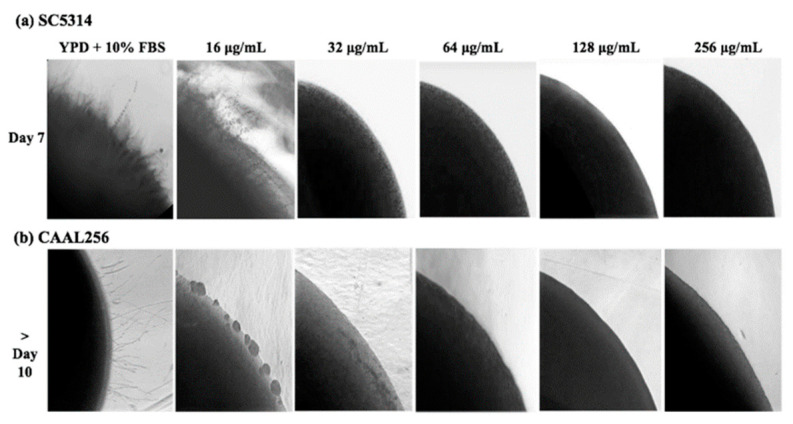
Effect of ethanolic extract of *P. nigrum* on filamentation of *C. albicans* on solid medium containing YPD plus FBS. *C. albicans* (**a**) SC5314 and (**b**) CAAL256 strains. Images were taken at 40× magnification.

The ability of the ethanolic extract of *P. nigrum* to disturb the hyphal formation of *C. albicans* was further evaluated by observing morphological changes of yeast colonies on YPD containing FBS supplemented with the ethanolic extract. In the reference strain *C. albicans* SC5314, the ethanolic extract of *P. nigrum* at ≥32 μg/mL, most of the colonies were completely smooth, and no filamentation was observed on the surface and edges; whereas the control colony contained witnessed filamentation (Figure 4). In good agreement with the inhibition of hyphae transition, the different extract concentrations also affected the colony morphology in the FLC-resistant clinical strain CAAL256.

### 3.4. Effect of P. nigrum on C. albicans Biofilm Formation

**Figure 5:**
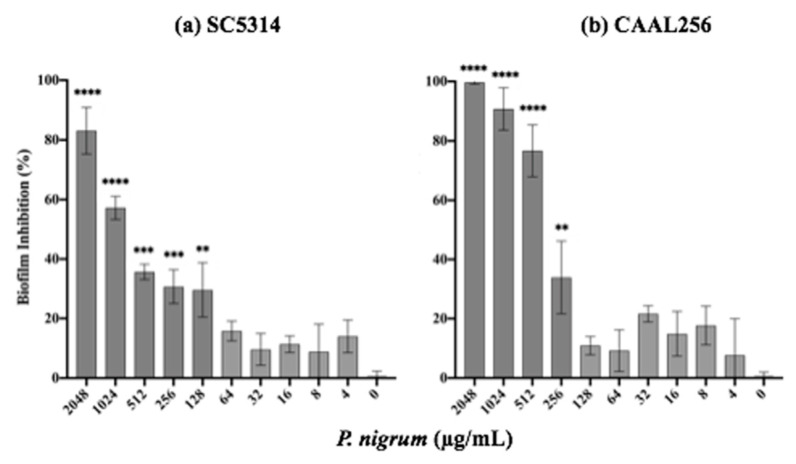
In vitro characterization of the inhibitory activity of *P. nigrum* ethanolic extract on *C*. *albicans* biofilm formation *p*-values of <0.05 were used to indicate statistical significance as follows: ** *p* ≤ 0.01. *** *p* ≤ 0.001. **** *p* ≤ 0.0001.

The ability to form biofilms represents a key virulence factor in *C. albicans*, and this process is closely related to filamentation. After 24 h of treatment with the ethanolic extract of *P. nigrum* on *C. albicans* strains (SC5314 and CAAL256), biofilm production was significantly affected in a concentration-dependent manner (Figure 5). In the strain SC5314, the ethanolic extract at concentrations of 128, 256, 512, 1024, and 2048 μg/mL inhibited it by about 30%, 31%, 35%, 57%, and 83%, respectively (Figure 5a). For the FLC-resistant CAAL256 strain, at concentrations of 256, 512, and 1024 μg/mL percentages of biofilm inhibition were about 33%, 76%, and 90%, respectively (*p* < 0.01) (Figure 5b). Treatment with caspofungin (control) completely inhibited biofilm formation in both strains at 0.125 μg/mL (data not shown).

### 3.5. Effect of P. nigrum on C. albicans Hydrolytic Enzyme Secretion

**Figure 6:**
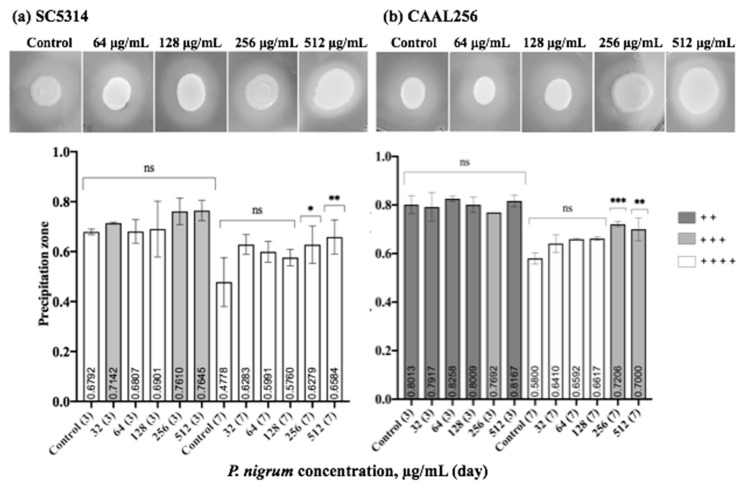
Phospholipase production by *C. albicans* (**a**) SC5314 and (**b**) CAAL256 strains. Control bars indicate untreated cells for 3 and 7 days. *Pz* classification: 1.0 (−) indicates that the tested strain was negative for phospholipase production: 0.99–0.90 (+) very low; 0.89–0.80 (+ +) low; 0.79–0.70 (+ + +) moderate producers; and *Pz* ≤ 0.69 (+ + + +) high producers. *p*-values of <0.05 were used to indicate statistical significance as follows: * *p* ≤ 0.05. ** *p* ≤ 0.01. *** *p* ≤ 0.001. ns: Not significant.

We further studied the effect of *P. nigrum* on phospholipase and proteinase production. In Figure 6, we summarize the phospholipase enzyme activity on strains in the presence and absence of sub-inhibitory concentrations of the ethanolic extract. After seven days of evaluation, both strains SC5314 (*Pz* < 0.47) and CAAL256 (*Pz* < 0.58) were phospholipase positive (high producers), being SC5314 the strain possessing the highest activity. After three days, the enzyme activity did not significantly change when the extract was used. Nevertheless, by day 7, at concentrations of 256 and 512 μg/mL, a significant change in phospholipase production was observed in both strains. However, there was no change in strain SC5314 in the categorical classification, i.e., high producer. In contrast, as for the strain FLC-resistant CAAL256, the use of the extract resulted in moderate phospholipase production.

**Figure 7:**
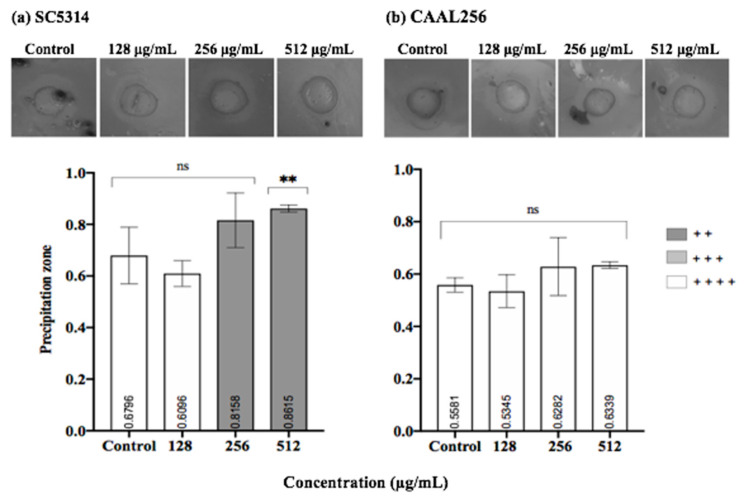
Proteinase production by *C. albicans* (**a**) SC5314 and (**b**) CAAL256 strains. *Pz* classification: 1.0 (−) non-producers when there was no visible lysis, 0.99–0.90 (+) very low, 0.89–0.80 (+ +) low, 0.79–0.70 (+ + +) moderate producers; and *Pz* ≤ 0.69 (+ + + +) high producers. **, *p* < 0.01, ns: not significant. The top panel shows pictures obtained when *C. albicans* strains were tested for proteinase activity in the absence (control) and presence of *P. nigrum* extract.

Considering proteinase activity, in the SC5314 strain, the ethanolic extract of *P. nigrum* at 512 μg/mL exhibited a significant reduction in proteinase production compared to the control (*p* < 0.01). A shift between high producer and very low producer was observed. (Figure 7a). However, when the CAAL256 strain was evaluated at concentrations of 128–512 μg/mL, there was no significant difference compared to the control (Figure 7b).

### 3.6. In Vitro and In Vivo Assessment of the Cytotoxicity of P. nigrum Extract

**Figure 8:**
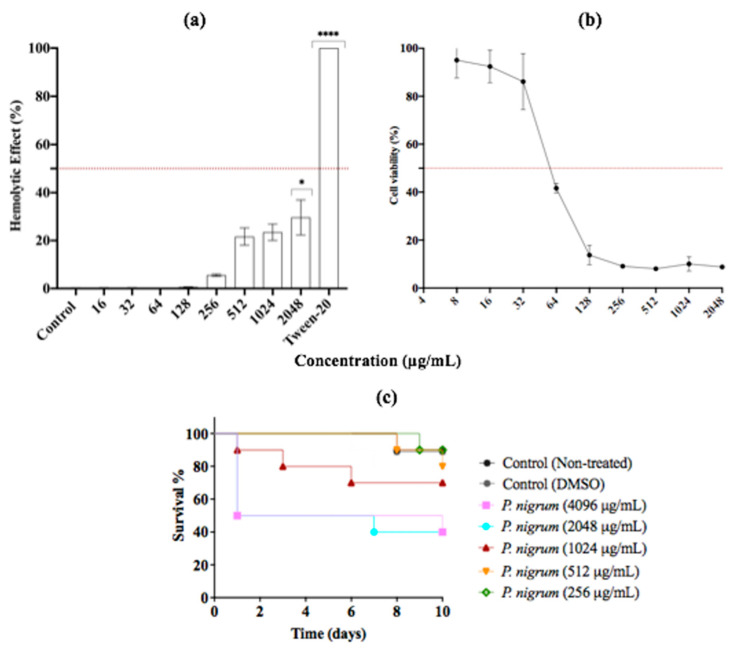
In Vitro and In Vivo Assessment of Cytotoxicity. (**a**) Human erythrocytes exposed to *P. nigrum* extract at 37 °C for 1 h. (**b**) Cell viability of Vero cells. (**c**) In vivo toxicity effect of treatment with *P. nigrum* extract on *G. mellonella* larvae. The data are expressed as the percentages of survival. Vehicle control: DMSO (<2%). *p*-values of < 0.05 were used to indicate statistical significance as follows: * *p* ≤ 0.05. **** *p* ≤ 0.0001.

Since adverse drug effects associated with antimicrobial use are a major concern, we evaluated first the hemolytic activity of *P. nigrum* ethanolic extract. The results showed that *P. nigrum* is safe and not toxic at a wide range of growth inhibitory concentrations (16–2048 μg/mL). Up to a concentration of 256 μg/mL, the extract showed a slight hemolytic effect on human red blood cells. At concentrations above 512 μg/mL, the hemolysis observed was moderate, with percentages of about 20–30% (Figure 8). Similar results were observed when the DCM fraction was used (Figure S4).

Regarding cytotoxicity in Vero cells, the IC_50_ and R^2^ values were 58.99 μg/mL and 0.96, respectively. The ethanolic extract showed no appreciable toxicity against Vero cells at 8–32 μg/mL, with percentages above 85% of cell viability. However, at concentrations above 64 μg/mL, the use of extract decreased the percentage of cell viability by more than 50%. Finally, *G. mellonella* was used as in vivo model to study the cytotoxicity of ethanolic extract of *P. nigrum*. After 10 days of monitoring, at concentrations of ≤1024 μg/mL, more than 70% of the larvae were alive. At higher concentrations, survival rates of around 40 and 50% were observed (Figure 8c).

**Figure 9:**
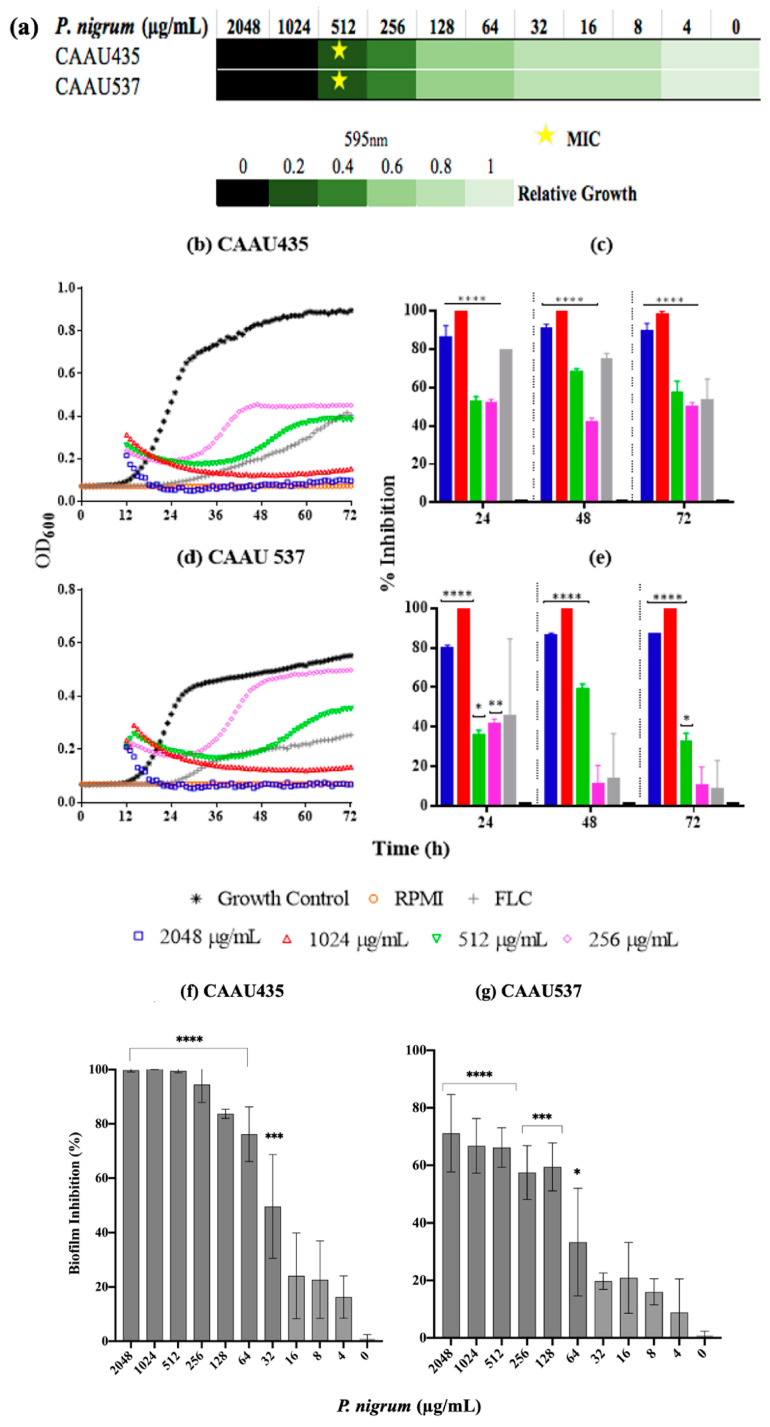
Anticandidal activity of *P nigrum* on *C. auris*. (**a**) *P nigrum* MICs (indicated by stars), the green bars indicate relative fold growth. (**b**,**d**) Killing kinetics curves (**c**,**e**) percentage of growth inhibition. (**f**,**g**) inhibitory activity against biofilm development. Control bar indicates untreated cells, accepted as 0% inhibition (0 μg/mL). *p*-values of <0.05 were used to indicate statistical significance as follows: * *p* ≤ 0.05. ** *p* ≤ 0.01. *** *p* ≤ 0.001. **** *p* ≤ 0.0001.

We also explored the activity of the ethanolic extract on *C. auris* growth and biofilm formation (Figure 9). The MIC and MFC values obtained after the exposure to *P. nigrum* (ethanolic extract) were 512 μg/mL for both strains (sensitive CAAU435 and FLC- AMB-resistant CAAU537), indicating a two-fold lower value than the one observed for the reference *C. albicans* strain. Using DCM and EtOAc fractions, the MICs observed were 2048 μg/mL (Figure S2). Concerning kinetic results, after 24 h of growth, the presence of the ethanolic extract (256–2048 μg/mL) significantly affected the growth in both *C. auris* strains. Inhibition values above 80% were observed at 512 μg/mL, data consistent with previous observations in the microdilution assay. Finally, the inhibitory effect of *P. nigrum* ethanolic extract on *Candida* biofilms was studied. *P. nigrum* significantly reduced biofilm formation at concentrations of ≥32 and 64 μg/mL in CAAU435 and CAAU537 strains, respectively (Figure 9f,g). The control (caspofungin) at 0.062 μg/mL significantly reduced biofilm formation in *C. auris* strains (data not shown).

## 4. Discussion

IFIs are responsible for killing more than 1.5 million people yearly, similar to mortality rates due to tuberculosis and about three times more than malaria [68]. The limited number of antifungal drugs available (i.e., polyenes, azoles, and echinocandins) and the spread of antifungal resistance amplifies the need to identify new fungal targets for developing novel therapeutic alternatives [58].

Various natural compounds with antibacterial and antiviral activity have been discovered [69]. However, the search for natural compounds with antifungal activity is less studied. Interestingly, *P. nigrum* has exhibited a broad spectrum of activity [46,47]. Therefore, we evaluated the antifungal activity of *P. nigrum* against clinical isolates of *C. albicans*. Herein, by BMD (gold standard method), the antimicrobial activity of the ethanolic extract of *P. nigrum* showed moderate antifungal activity against *C. albicans* and *C. auris* strains. Although DCM and EtOAc fractions were also evaluated, the ethanolic extract was more effective. The antifungal activity observed might be attributable to the presence of secondary metabolites corresponding to alkaloids, tannins, and terpenoids found in the qualitative phytochemical test. According to recent reports, the ethanolic extract of *P. nigrum* showed antifungal activity with MIC values between 500–2000 μg/mL against reference strains of *C. albicans* using the disc diffusion technique [48]. Our results are comparable with studies that have demonstrated the antibacterial and antifungal activity of the ethanolic extract of *P. nigrum* against clinically important pathogens such as *E. coli, S. aureus,* and *C. albicans* [70,71]. Furthermore, some studies have shown that the ethanol fraction is often the most effective [72,73]. However, the strongest antimicrobial activity has been observed in an acetonic extract (*Cinnamomum zeylanicum bark*) against *C.albicans* [74]. In bacteria, the study of Ganesh et al. showed that a chloroform extract of *P. nigrum* displayed higher antibacterial activity than the ethanolic extract against *E. coli*, *Salmonella typhi*, *Proteus* sp. [75]. Nevertheless, the variation in MIC values could be due to the method used, as well as the testing against drug-resistant strains.

*P. nigrum* contains a bioactive compound known as Piperine, an alkaloid belonging to the alkamide family [76]. Among the alkamides isolated from *P. nigrum,* Piperine, Piperettine and Piperettyline have been reported to have antimicrobial, antioxidant, and anti-inflammatory properties [77,78]. To give an approximate identification of the possible lead compound present in *P. nigrum* fruit, an MS analysis was performed to determine the fragmentation patterns of the major peak found. LCMS analysis showed the presence of several characteristic fragmentation patterns as *m/z* 286 ([M + H]^+^) with specific fragments of *m/z* 201 ([M–C_5_H_11_N]^+^), 171 ([M–C_6_H_13_NO]^+^), 143 ([M–C_7_H_13_NO_2_]^+^) and 115 ([M–C_8_H_13_NO_3_]^+^. Thus, based on its chromatographic analyses, mass spectrum, and fragmentation route, the major peak of the DCM fraction corresponds to Piperine (Figure S5) as previously described [77,79,80]. Nonetheless, it is important to emphasize that the isolation process must continue to determine the compounds responsible for the activity.

As virulence traits are pivotal in pathogenicity and have been considered a possible antimicrobial target [81], we studied the inhibitory effect of *P. nigrum* in some of the most important virulence factors of *C. albicans*.

It is well recognized that the morphological transition of *C. albicans* can influence its pathogenicity. Both blastoconidia and hyphae play a crucial role in pathogenesis; however, these display different functions, which include adhesion and invasion [82]. Several studies have focused on inhibiting these morphological transitions in pathogenic fungi; nevertheless, there is scarce knowledge about the action of natural compounds against this virulence trait in *C. albicans* [83,84,85,86,87,88,89]. Some natural and synthetic compounds have been tested and found to be effective in affecting germ tubes or pseudo-hyphae in *C. albicans* [77,80,84,85]. However, to our knowledge, there is no scientific evidence of the role of *P. nigrum* extract in the yeast-to-hyphae transition. An important clue was previously observed by using Piperine (the major component in *Piper* species) since it impeded biofilm formation and hyphal morphogenesis in *C. albicans* [34]. Herein, using two hyphal-inducing media, we observed that the hyphal growth of *C. albicans* strains was repressed by the *P. nigrum* extract. This is an encouraging result, as this extract can affect one of the most important virulence factors of *C. albicans,* even in resistant strains. Yet, the pathogenicity profile is not necessarily related to hyphal formation; therefore, filamentation damage and other virulence factors could be even more promising [90]. Regarding the anti-biofilm activity, it was also possible to affect the biofilm formation in both *C. albicans* strains, which was an expected result considering the anti-filamenting activity observed. Similar results had been described against *C. albicans* ATCC 90028, with extracts derived or even metabolites from the same genus of the plant used in this study [91].

In addition to the hyphae and the biofilm formation, *C. albicans* secretes hydrolytic enzymes that favor infectious processes and improve the acquisition of extracellular nutrients. Within these hydrolases, *C. albicans* secretes three classes: proteases, phospholipases, and lipases [16]. Proteases have been considered to contribute to colonization, tissue penetration, and evasion of the immune response after the degradation of host defense proteins. Although some studies evidence that aspartic proteases (Saps) are related to the pathogenicity of *C. albicans,* this is still a controversial issue [16,62]. In the case of phospholipases, they play an important role in tissue invasion by disrupting the cell membranes of epithelial tissue [62]. In contrast to the previous hydrolases, lipases have been the less studied [24]. For that reason, hydrolytic enzymes, especially proteinases and phospholipases, are a study target for developing new therapeutic treatments. In this study, the ethanolic extract of *P. nigrum* decreased hydrolytic enzyme secretion in *C. albicans* strains. Of note, even if there are studies where the inhibition of secretion of hydrolytic enzymes with natural products was proved [92,93], this is the first study that evaluates the anti-enzyme activity of extracts from *P. nigrum* against *C. albicans* strains that are sensitive and resistant to antifungals. This is an interesting finding, considering the ability to affect another important virulence factor in *C. albicans* [94].

The correlation between hydrolytic enzyme secretion and antifungal resistance in *Candida* spp. is controversial, as few studies have investigated this issue in depth. Silva et al. suggested an increased expression of SAP genes in yeasts exposed to antifungals at sub-inhibitory concentrations or with intrinsic resistance. However, Seifi et al. did not find a significant relationship between extracellular enzyme levels and sensitivity to FLC [95,96].

Here, both the FLC-sensitive strain SC5314 and the FLC-resistant strain CAAL256 produced high levels of hydrolytic enzymes. However, in terms of phospholipase production, although both strains are producers, SC5314 is a higher producer than the resistant strain. This indicates that there is no direct correlation between secretion of these enzymes and FLC resistance in these strains; therefore, further studies with larger numbers of sensitive and resistant clinical isolates are warranted.

Studies using only reference strains are common and certainly relevant. Nevertheless, clinical strains are desirable to observe the diverse phenotypes and responses by strains that can grow under stressful conditions. Furthermore, antifungal resistance involves the regulation of several signaling pathways associated with drug response (e.g., Ras/cAMP/PKA pathway, calmodulin/calcineurin pathway (CaM/CaL), and mitogen-activated protein kinase [MAPK] signaling pathway). These pathways may favor not only resistance but also pathogenicity, so it is possible to observe different phenotypes across isolates [58]. However, these complex and multifactorial processes need to be studied in depth.

On the other hand, the pharmacological effects associated with the use of antifungals are a major concern due to the eukaryotic nature of the target organism (yeast) and the host. Thus, it is imperative to evaluate the toxicity of the plant extracts [97]. Here the assessment of hemolysis suggests that even at high concentrations of *P. nigrum* use, low percentages of hemolysis were induced. Conversely, cell viability in Vero cells was affected when the extract was used at low concentrations. Notably, the crude extract was not cytotoxic when used at a concentration that inhibited germ tube and hyphal growth in *C. albicans* (i.e., 8 to 32 μg/mL). Studies have reported that many plant extracts contain saponins, a hemolytic compound [98]. Herein, the phytochemical analysis revealed that *P. nigrum* extract and its DCM fraction do not contain this phytochemical, suggesting its low hemolytic activity. However, toxicity in Vero cells deserves further study to determine the compound causing the toxicity and to improve this issue [99,100]. Interestingly, the in vivo test in the larvae model evidenced low toxicity (80% of survival) at an extract concentration of less than 512 μg/mL. Hithereto, only one study has revealed that Piperine, the alkaloid detected in Piper species, does not display a toxic effect in *Caenorhabditis elegans* [34].

Although *C. albicans* remains the predominant cause of IFIs, other *Candida* species have emerged, such as *C. auris,* which can cause invasive infections and is considered a multi-drug resistant species, the latter being a major concern diminishes treatment options [101]. In this scenario, our results are encouraging since *P. nigrum* exerted antifungal activity against the human threat *C. auris* [102]. To our knowledge, this is the first study that described the activity of *P. nigrum* against this fungus; however, complementary studies are needed to determine the spectrum of activity.

Taking into account the importance of biofilm formation, the results obtained are also relevant [103,104] since yeast biofilms can be 100 times more resistant to fluconazole and 20 to 30 times more resistant to amphotericin B compared to planktonic cells [105,106].

Overall, *P. nigrum* extract affected *Candida* growth and morphogenesis, demonstrating that this natural product has a non-negligible anti-candida activity and represents a promising resource for discovering novel antifungal compounds. Nevertheless, a considerable amount of research remains to be conducted, such as determining the mechanism of action. Some authors suggest that the fungicidal effect of *P. nigrum* may be caused by the breakage of glucans and/or cell wall lysis.

## 5. Conclusions

*P. nigrum* affected hyphal development, as well as phospholipases and proteinases activity in *C. albicans*. Furthermore, the ethanolic extract of *P. nigrum* had activity against biofilms in *C. albicans* and *C. auris,* even in drug-resistant strains. In terms of toxicity, interesting results were found as the *P. nigrum* extract showed no hemolytic effect in vitro and exhibited reduced cytotoxicity on Vero cells and *G. mellonella* larvae at concentrations that inhibited hyphae and biofilm in *C. albicans*. This plant represents a promising source for discovering novel antifungal compounds concerning the emergence of antifungal resistance and the low number of available antifungal drugs.

## Data Availability

Not applicable.

## Supplementary Materials

The following supporting information can be downloaded from the following link: https://www.mdpi.com/article/10.3390/jof8080784/s1, Figure S1: Chromatographic elution by HPTLC for alkaloids and tannins from *P. nigrum* fruits extract. Figure S2: Anticandidal properties of *P. nigrum* extract fractions. The fractions such as Hex (n-hexane), DCM (dichloromethane), EtOAc (ethyl acetate), and EtOH (ethanolic extract) were evaluated by triplicate in *C. albicans:* SC5314 and CAAL256 and *C. auris* CAAU435 and CAAU537 strains and incubated at 37 °C for 48 h. Figure S3: Effect of crude ethanol extract fractions treatment on phenotypic switching. Percentage of filamentation in standard strain *C. albicans* SC5314 in the absence (Control) and presence of different concentrations of extract fractions under hyphae-inducing conditions. Figure S4: Hemolysis assay of DCM fraction of *P. nigrum* extract. Figure S5: Piperine mass fragments: possible structures of major mass spectral fragments of piperine.
