# Supplementary material for: Evaluation of Anti-Candida Potential of Piper nigrum Extract in Inhibiting Growth, Yeast-Hyphal Transition, Virulent Enzymes, and Biofilm Formation
Source: J Fungi (Basel). 2022 Jul 27;8(8):784. doi: 10.3390/jof8080784 (PMC9409899; doi:10.3390/jof8080784)
Supplement: Supplementary file 1 [file jof-08-00784-s001.zip › jof-1806044-supplementary.pdf]

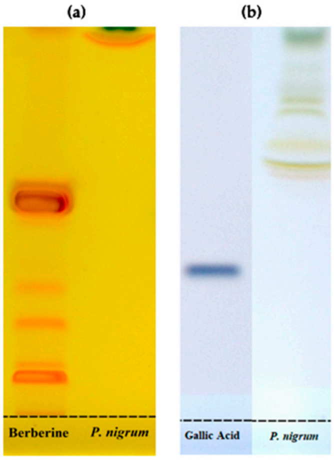

Figure S1. Chromatographic elution by HPTLC for alkaloids and tannins from *P. nigrum* fruits extract. (a) SP: HPTLC Silica gel 60 F_{254}; MP: Toluene: EtOAc: Diethylamine 70:20:10 (*v*/*v*/*v*); Rev.: Dragendorff VIS Reagent; C+: berberine. (b) SP: HPTLC Silica gel 60 F254; MP: Toluene:EtOAc:MeOH:Formic Acid 75:25:25:6 (*v*/*v*/*v*/*v*); Rev.: Reagent FeCl3 at 1% VIS.

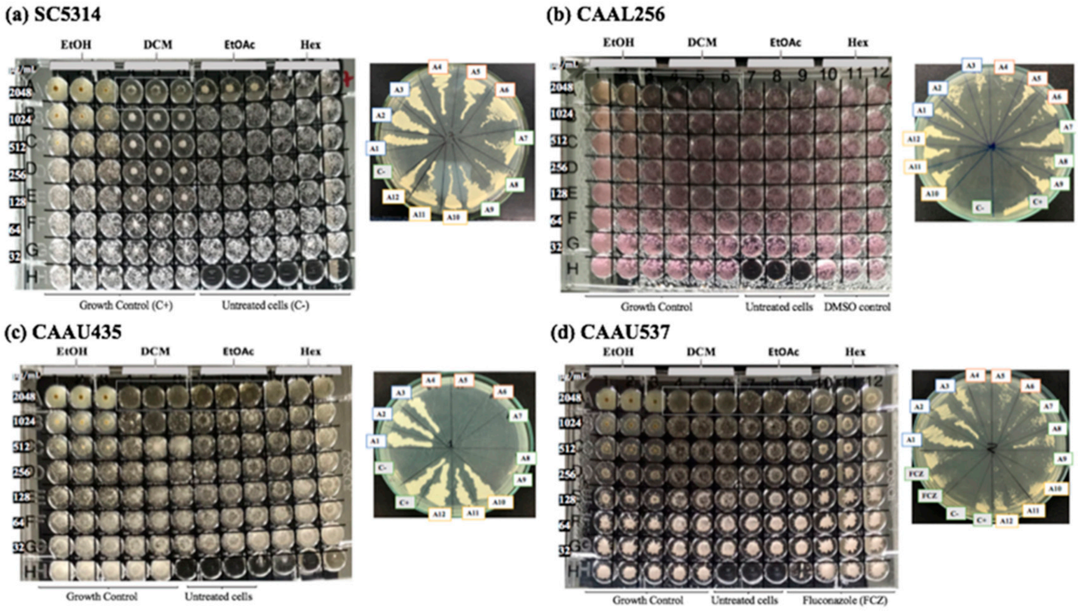

Strains	MIC (µg/mL) EtOH:H2O	MFC (µg/mL) EtOH:H2O	MIC (µg/mL) DCM	MFC (µg/mL) DCM	MIC (µg/mL) EtOAc	MFC (µg/mL) EtOAc	MIC (µg/mL) Hex	MFC (µg/mL) Hex
C. albicans								
SC5314 (WT)	>2048	>2048	>2048	>2048	>2048	>2048	>2048	>2048
CAAL256	2048	>2048	2048	>2048	>2048	>2048	>2048	>2048
C. auris								
CAAU435	>2048	>2048	2048	2048	>2048	>2048	>2048	>2048
CAAU537	>2048	>2048	2048	2048	2048	2048	>2048	>2048

Figure S2. Anticandidal properties of *P. nigrum* extract fractions. The fractions such as Hex (n-hexane), DCM (dichloromethane), EtOAc (ethyl acetate) and EtOH:H_2O (ethanolic extract) were evaluated by triplicate in *C. albicans (* SC5314, CAAL256), and *C. auris* (CAAU435, CAAU537) strains, and incubated at 37 °C for 48 h. The MIC_{80} was determined as the concentration where the viability of the cells was reduced by 80 % as compared with the growth control. C-: untreated cells; C+: growth control and DMSO (0.5, 1, and 2), FLC (32, 64, and 128 µg/mL).

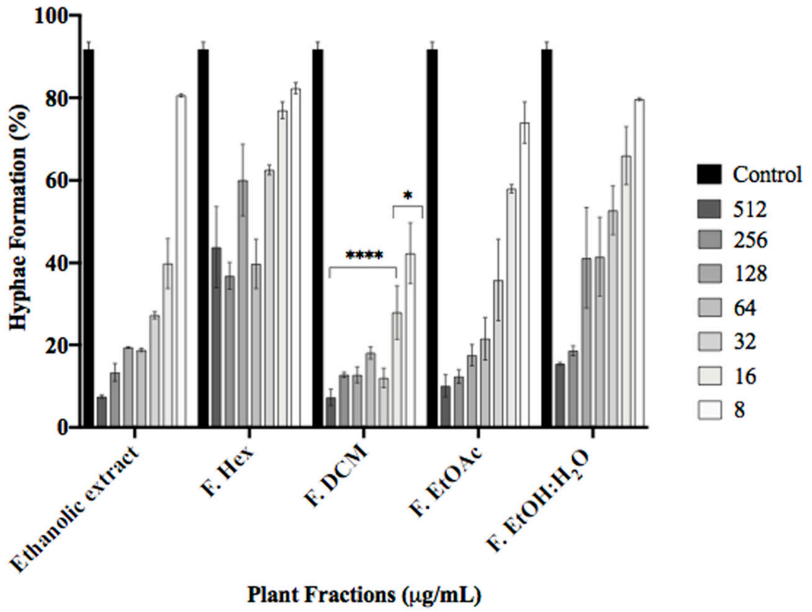

Figure S3. Effect of crude ethanol extract fractions treatment on phenotypic switching. Percentage of filamentation in *C. albicans* SC5314 in the absence (Control) and presence of different concentrations of extract fractions under hyphae-inducing conditions. F. Hex: n-hexane fraction, F. DCM: dichloromethane fraction; F. EtOAc: ethyl acetate fraction and F. EtOH:H₂O: ethanolic fraction. Data represent the means ± the standard deviations of two independent experiments. *$p < 0.05$; **** $p < 0.0001$ (2way Anova, and Dunnett multiple comparisons test).

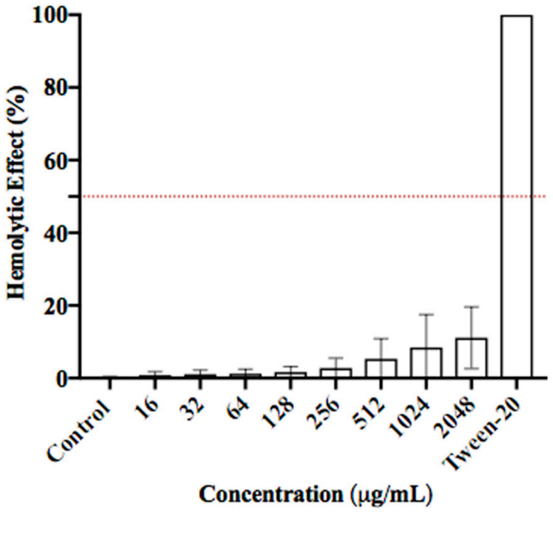

Figure S4. Hemolysis assay of DCM fraction of *P. nigrum* ethanolic extract. A 96-well plate for hemolysis of human erythrocytes exposed to DCM fraction, ranging from 16 to 2048 µg/mL at 37 ºC for 1 hour. The experiment was conducted in triplicate. The negative control was a measure of spontaneous hemolysis and contained centrifuged erythrocyte suspension in PBS. Positive control: tween 20.

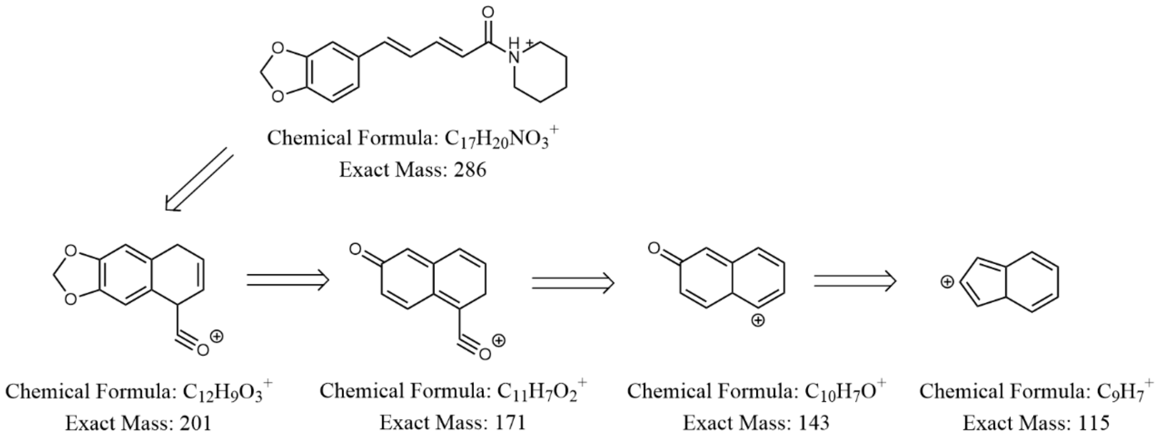

Figure S5. Fragmentation pattern in mode positive of the major peak 1 present in the DCM fraction. Adapted from Kotte et al., [79].
